# An Automatic User Activity Analysis Method for Discovering Latent Requirements: Usability Issue Detection on Mobile Applications

**DOI:** 10.3390/s18092963

**Published:** 2018-09-05

**Authors:** Soojin Park, Sungyong Park, Kyeongwook Ma

**Affiliations:** 1 Graduate School of Management of Technology, Sogang University, 35 Baekbeom-ro, Mapo-gu, Seoul 04107, Korea; 2 Department of Computer Science and Engineering, Sogang University, 35 Baekbeom-ro, Mapo-gu, Seoul 04107, Korea; parksy@sogang.ac.kr (S.P.); kyeongwook.ma@gmail.com (K.M.)

**Keywords:** internet of things, activity modeling, context-awareness, self-adaptive systems, usability, mobile application

## Abstract

Starting with the Internet of Things (IoT), new forms of system operation concepts have emerged to provide creative services through collaborations among autonomic devices. Following these paradigmatic changes, the ability of each participating system to automatically diagnose the degree of quality it is providing is inevitable. This paper proposed a method to automatically detect symptoms that hinder certain quality attributes. The method consisted of three steps: (1) extracting information from real usage logs and automatically generating an activity model from the captured information; (2) merging multiple user activity models into a single, representative model; and (3) detecting differences between the representative user activity model, and an expected activity model. The proposed method was implemented in a domain-independent framework, workable on the Android platform. Unlike other related works, we used quantitative evaluation results to show the benefits of applying the proposed method to five Android-based, open-source mobile applications. The evaluation results showed that the average precision rate for the automatic detection of symptoms was 70%, and the success rate for user implementation of usage scenarios demonstrated an improvement of around 21%, when the automatically detected symptoms were resolved.

## 1. Introduction

Mobile Edge Computing (MEC), Cyber-Physical Systems (CPS), and other applications recently developed based on the Internet of Things (IoT), are perceived as promising next-generation paradigms [1,2,3]. One of the elements that typically characterizes such new software system paradigms is autonomy. Individual systems participating in IoT-based collaborations, have the autonomy to detect and respond appropriately to environmental changes that occur during service execution [4]. Change requests explicitly made by users can be controlled by the change management procedure, even in the traditional software engineering process. Unexpected environmental changes in IoT-based collaborations sometimes cause “latent requirements”, meaning unmet needs the user finds difficult to express explicitly. Although an initially provided system may meet all requirements, diverse environmental changes can produce greater numbers of latent requirements as system runtime increases. In the worst-case scenario, system users may feel uncomfortable and leave the system, rather than issuing an explicit change request.

To solve this problem, this paper proposes an automatic user activity analysis method for discovering latent requirements. Latent requirements occur when there exists a difference between an expected usage model and the user’s actual application usage. For this, an action log template is used to capture valuable information from interactions between a user and a system; an activity model that can be extracted from captured action logs to represent a user’s behavior; and an instantiated rule, set for detecting bad symptoms. The automatic user activity analysis method proposed in this paper involves three steps, from capturing action logs to discovering bad symptoms, leading to latent requirements. First, interactions between a user and a system are captured, and valuable information is saved according to the action log template. The accumulated action logs are periodically converted into a user activity model. In the same way, an expected activity model is automatically generated from the developer’s usage of the application. Second, multiple activity models from diverse users are merged into a single activity model to represent aggregate user behavior. Third, a comparison is made between the merged user activity model and the expected activity model. The differences between the two activity models are analyzed by predefined rules for detecting bad symptoms that hinder a specific quality attribute.

In addition to our previous work [5], which proposed only a stand-alone tool for the automatic detection of usability issues on mobile applications, this paper extended the bad symptom detection mechanism of the tool to a self-adaptive framework, applicable to all applications running on the Android platform. In general, the self-adaptive framework not only detects environmental changes and latent requirements on running mobile applications, but also supports a complete self-adaptation cycle, from establishing and implementing a configuration change plan, to resolving the detected latent requirements. However, the self-adaptive framework used in this paper, refers only to the local adaptation part of the reference framework supporting the IT ecosystem [6]. The feasibility of the proposed framework was shown via a prototype that automatically resolved a latent requirement on the GUI design of a shopping mall application running on the Android platform. Usability was selected as the quality issue subject for automatic detection, because users are more sensitive to usability than to other quality attributes of mobile applications [7]. 

To evaluate the accuracy and benefits of the proposed usability issue detection method, five Android-based open-source mobile applications were used to find latent requirements. Accuracy was measured by comparing the issues reported to the actual open-source community, with the automatically detected bad symptoms. In the five mobile applications, on average, 70% of the automatically detected bad symptoms were consistent with the usability issues reported in the open-source community. To demonstrate another benefit in this study, we measured average goal achievement rates by usage scenario and average elapsed time to achieve a goal before and after resolving the automatically detected bad symptoms. As a result, the average goal achievement rate increased by 21%, while the average elapsed time to achieve a goal decreased by 1082.4 ms. Considering that most evaluations on the usability of mobile applications are limited to applications and scenarios developed in-laboratory, our quantitative evaluations yielded encouraging results.

The composition of the paper is as follows. Section 2 reviews related works, while Section 3 introduces the overall framework supporting the proposed automatic user activity analysis. Section 4 presents an overview of the working mechanism of the automatic user activity analysis method introduced in Section 3, whilst Section 5 describes the activities of the proposed method step by step. Section 6 discusses the application results of the proposed method for several open-source mobile applications, to determine the effectiveness of the proposed framework and method. Lastly, Section 7 presents the conclusions of the study and future study plans.

## 2. Related Work

### 2.1. Usability Testing Method for Mobile Applications

Existing studies on the usability of applications, particularly the usability of GUIs, have largely relied on heuristic methods. The proposals in References [8,9] were based on questionnaire surveys, which directly asked users questions related to usability, to measure and improve the usability of systems. In line with efforts to ensure the objectivity of application usability evaluations based on questionnaire surveys, Reference [10] presents the results of experiments in which goal-question metrics were defined to measure both the time taken for a user to implement a certain scenario and the degree of a user’s context awareness during application usage. However, utilizing user questionnaires as the primary method to gather basic usability data may yield inconsistent results, depending on the organization of questionnaire items, the characteristics of respondents, and other variables. On the other hand, the A/B test for usability evaluation presented in [11] provides users with the same page/content, whilst modifying the concrete format or design of messages to gauge individual users’ responses. Although this method ensures some objectivity by providing uniform content to users, it is constrained by the potential for rapid increases in the time and resources required for usability testing. Specifically, because individual users’ responses to diverse GUI alternatives should be evaluated, the method requires the fabrication of a prototype for each of these alternatives. 

In addition to questionnaires on the usability of mobile application GUIs, some studies have focused on aesthetics [12]. Such studies have argued that the degree to which users feel comfortable using a certain application can be measured by, for example, the degree of alignment of mobile application screen views or the width and height of widgets. However, given that mobile applications operate in heterogeneous device environments, even for the same mobile application, GUI evaluation results may differ due to varying device resolution settings. Proposing an approach to evaluate early usability based on model-driven architecture (MDA), Reference [13] defined a usability model framework that included the platform-independent model (PIM) and the platform-specific model (PSM). The proposed framework was used to evaluate and improve the usability of a system at the PIM level, even without a final version of the user interface, and the results of applying the proposed framework to an MDA tool were discussed.

The study in [14] deviates from the usability evaluation method, centered on the analysis of questionnaire results, adopting a method of objectively monitoring a mobile application. It proposed a separate model to evaluate the usability of smartphone applications and an infrastructure that supported implementation of the proposed evaluation model. Unlike previous studies, it presents workable models to automatically monitor an Android-based mobile application and discusses the results of applying several usability-related metrics to field experiments, instead of laboratory-controlled experiments. However, as with previous studies, it involved users directly answering questions related to the sense of usability felt whilst using the application. 

The latest studies in [15,16,17,18] have proposed automatic usability testing methods, based on event logging. They mainly use the automated data collection method and analyze experimental results from laboratory and/or from remote users. The target software of the usability evaluation in [15,16] was not mobile application, but web-based application. The usability testing result shown in [17] was also focused on the mobile websites, rather than the mobile applications. From a research perspective, the method proposed in [18] was similar to our proposed method in that they automatically collect user interface events on Android-based mobile applications, and compare state-machine based models derived from users’ and experts’ event logs. However, the differentiation point of our work is on the contents of the automatically produced usability evaluation result. The usability testing results from [18] were quite fragmentary information. Thus, in order to answer what the usability issues of the present system are, additional analysis of the expert on the automatically generated usability testing result is required. Since our automatic latent requirements detection method was developed in line with the self-adaptive framework development, it was designed with the goal of self-adaptation of the software itself to resolve a large part of the automatically detected usability issues. Therefore, the automatically detected usability issues by our proposed method are directly explaining what problems are latent in a mobile application, without any further interpretation.

### 2.2. Self-Adaptive Software Framework

Some noticeable studies of frameworks that support self-adaptive software systems, include Rainbow [19], MUSIC [20], and DiVA [21]. All of these frameworks have been proposed to realize the MAPE-K loop. The frameworks make the software itself collect and analyze environmental changes detected at running time, from inside and outside of the software system. They also make the self-adaptive software system appropriately responds to the detected environmental change, if it has a meaningful context. 

The Rainbow framework secures its own originality as the first framework that systematically suggests a reusable infrastructure, which separates adaptation logics from application logics. Since Rainbow has been introduced, many studies have proposed various types of adaptive software with reference to the Rainbow framework. Since Rainbow defines a reusable infrastructure, it has the advantage of being able to achieve self-adaptation with relatively little expense and effort. On the other hand, there are limitations that only behavior rules specified in advance are operable. MUSIC offers a solution that combines a component-based development approach with a service-oriented architecture (SOA), to address the dynamic changes in mobile environments. It classifies all the required components into categories, such as business logic, context awareness, and concern of adaptation. Subsequently, it solves adaptation issues by combining the most appropriate components for a given situation. In some cases, however, the adaptation plan must be manually updated or replaced, since the adaptation goal model is not presented. DiVA focuses on providing a framework for developing a self-adaptive system and managing the variability of a self-adaptive system, rather than suggesting an infrastructure framework. The architecture of DiVA is based on aspect-oriented programming (AOP), and it supports self-adaptation in a way that dynamically adds the necessary aspects in the form of plug-ins.

Although the mentioned research results have their own advantages and disadvantages, the targeted systems are limited in a single system and their studies are focusing on local adaptation mechanisms occurring within a single system. To support IoT-based collaborations of autonomous devices, the targeted application domain should be extended to the self-adaptation of multiple systems. Case studies covering various service domains in the fields of system of systems (SoS) and CPSs are being published. However, until now, conceptual prototyping cases have been mainly presented on, and research results applicable to our daily life are few. The self-adaptive framework proposed in this paper supports the self-adaptation from automatic detection to solving the usability issues of mobile applications. We can get the benefits from this study in our daily life, though its application field is not extensive. Furthermore, the benefits are quantitatively evaluated by the experiments on mobile applications in the field, and not on in-laboratory applications.

## 3. A Self-Adaptive Framework for Automatic User Activity Analysis

In this section, a self-adaptive framework that supports automatic user activity analysis, for the detection of latent requirements is introduced. Then, the automatic user activity analysis method itself is presented. In our previous study [6], we proposed a framework for orchestrating context-aware IT ecosystems, highlighting a mechanism for selecting an optimal collaboration configuration among multiple autonomous devices. The previously proposed framework also supports a local adaptation mechanism, which detects environmental changes perceived in a single device and supports appropriate adaptative responses to these changes. To develop a workable architecture to realize the proposed automatic user activity analysis method on the Android platform, we referenced part of the framework in Reference [6] for local adaptation mechanisms.

**Figure 1:**
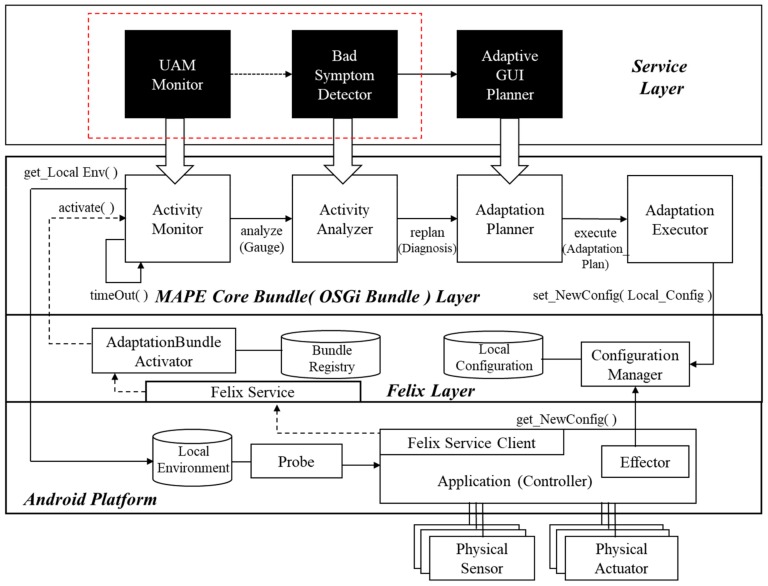
A self-adaptive framework for automatic user activity analysis.

To construct a new framework for the proposed method, we instantiated the framework in Reference [6] by defining several new problem-specific components. Figure 1 shows the instantiated framework, with the following running mechanism: When an application running on a mobile phone equipped with an Android platform is run, *AdaptationBundleActivator*, which belongs to the *Felix* layer, looks up *Bundle Registry* to dynamically bind bundles that fit three of the four bundles (excluding the *Adaptation Executor*) located in the *MAPE Core Bundle Layer* supported by OSGi [22]. The *ActivityMonitor*, *ActivityAnalyzer, AdaptationPlanner*, and *AdaptationExecutor* bundles defined in the *MAPE Core Bundle Layer*, combine to become a self-adaptive framework to drive a series of MAPE (Monitor-Analysis-Plan-Execute) [4] cycles to monitor and analyze user activities, as well as to establish and execute appropriate adaptation plans when problems are found. These four components defined in the *MAPE Core Bundle Layer* define generic roles, for running a general MAPE cycle. Therefore, to implement a MAPE cycle for achieving a certain goal, three components belonging to the *Service Layer*, defined according to each adaptation’s purpose, are dynamically bound to each of the three components in the *MAPE Core Bundle Layer* when the mobile application is initiated. *AdaptationExecutor* is an instantiated component that already implements an execution mechanism for dynamic reconfiguration, whilst the other three components of the *MAPE Core Bundle Layer* are stubs that do not involve actual implementations. Dynamic reconfiguration plans vary depending on the application domain, but the mechanism for executing a given dynamic reconfiguration plan is identical, regardless of the domain. For this reason, only the three components belonging to the *Service Layer* are dynamically bound to the components in the *MAPE Core Bundle Layer.*

Among the various quality dimensions of user activity analysis, usability was chosen for this study. *UAMMonitor*, *BadSymptomDetector*, and *AdaptiveGUIPlanner,* defined in the *Service Layer,* are OSGi bundles that generate user activity models in real time, use the models to detect “bad” symptoms that hinder usability, and establish adaptation plans to address these symptoms. The initiation phase is completed when each of the *Service Layer* bundles has been bound to its respective *MAPE Core Bundle Layer* bundle. If a user touches certain coordinates of a mobile phone, *Probe,* ported onto the Android platform, intercepts the event-related information and stores it in *Local Environment* storage as a log. This log includes the user’s touch coordinates, time stamps, gesture types, and so forth, collected using the Android macro, Monkeyrunner [23]. Information about screen configuration, including identification (ID) values of individual GUI screens, is extracted using the Android Hierarchical Viewer [24], and stored in the form of XML in *Local Environment* storage. 

The *UAMMonitor* periodically activates the *get_LocalEnv( )* function to fetch information regarding user actions and transform it into an activity model in the form of a state diagram in unified modeling language (UML). The generated activity model is handed over to the *BadSymptomDetector*, and compared with a predefined rule set to identify whether bad symptoms exist in terms of usability. When a bad symptom is detected, the *AdaptiveGUIPlanner* creates an adaptation plan, including changes to the GUI configuration, to resolve it. The newly created adaptation plan is saved in *Local Configuration* storage by the *ConfigurationManager*, while the *Effector* (which has been ported onto the application) requests a new configuration from the *ConfigurationManager,* and then changes the actual GUI configuration after receiving it.

**Figure 2:**
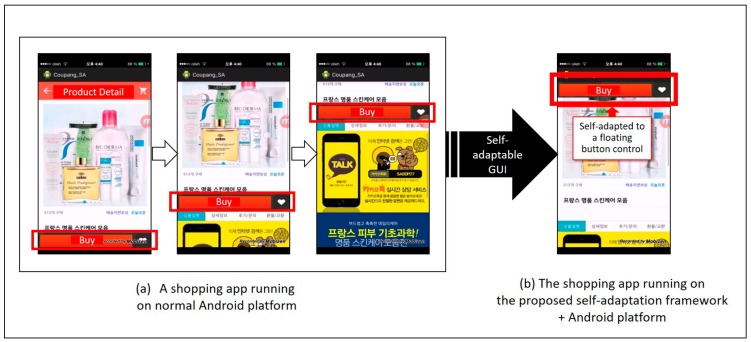
Comparison of the same mobile app (**a**) on a normal Android platform; and (**b**) on the proposed self-adaptation framework.

Figure 2 depicts the difference between running a real shopping mall (in Korea) app on a regular Android phone and running it on an Android phone with the self-adaptive framework, introduced in Figure 1. In this example, as described in Figure 2a, a user swipes down on the screen to view detailed information on a product. If the user wants to purchase the product, he or she must swipe up again and click the purchase button. In this process, the *BadSymptomDetector* can capture the following activity pattern: the *BuyButton.click( )* event follows several repeated *BuyButton.slideDown( )* and *Buy Button.SlideUp( )* events from activity models collected from multiple users. This kind of bad symptom is classified as a REPEATED_GESTURE symptom by the *BadSymptomDetector.* After bad symptom analysis, *AdaptiveGUIPlanner* selects a strategy from among the pre-defined self-adaptation rules or infers a new strategy from them. In this case, the selected plan is to change the buy button to a floating control on the view for users to directly access, without repeated swiping up and down. Figure 2b shows the automatically changed GUI design, completed by the proposed self-adaptive framework.

As shown in Figure 2, we have already developed several prototypes for self-adaptable GUIs. However, we have yet to determine the complete rules or inference methods to make a proper adaptation plan, and to develop a general mechanism for executing a self-adaptive reconfiguration plan. Thus, we limited the scope of this paper to the automatic detection of latent requirements through user activity analysis. As highlighted by the box with red dotted lines in Figure 1, the remainder of this paper focuses mainly on the latent requirements detection method, implemented by the *UAMMonitor* and the *BadSymptomDetector*.

## 4. An Overview of the Automatic User Activity Analysis Method

This study defines the root causes of latent requirements as any “gap” between the system behavior as currently implemented, and the system behavior that users desire. The gap can be detected by analyzing the actions that users perform on the system. A simple illustrative example from daily life demonstrates this process: When a bottle lid is manufactured to open in a clockwise direction, the user may show that the bottle lid is perceived as abnormal by repeatedly taking the action of turning the lid counterclockwise. In such a case, we can analyze the user’s repeated effort to turn the lid counterclockwise without achieving the purpose of opening the lid, in order to detect that the bottle lid was not implemented to satisfy the user’s desire. From this analysis, the producer of the bottle lid can understand that the currently implemented design has a problem and fix it in the next production cycle.

**Figure 3:**
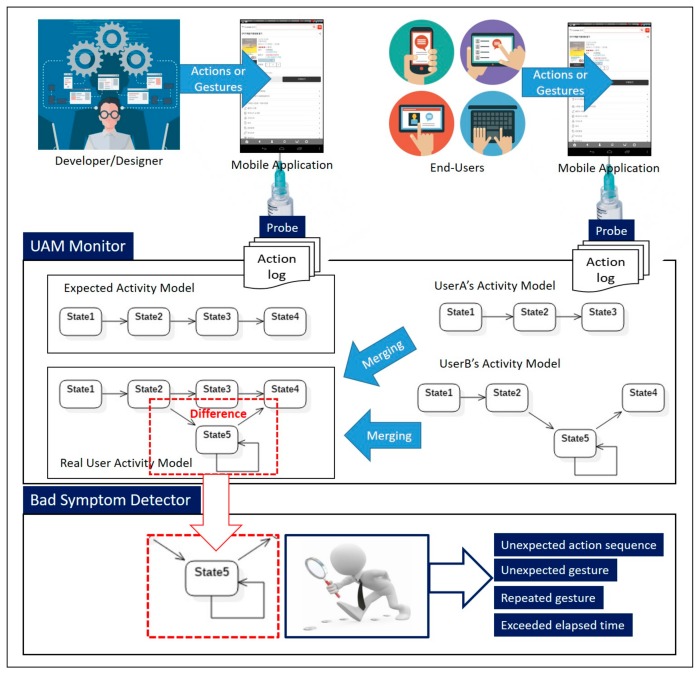
Overview of automatic user activity analysis method.

In this section, an overview of the proposed automatic user activity analysis method is presented in Figure 3, and then discussed. At first, system designers elicit and analyze initial requirements collected from users. The results of this analysis are then reflected in the system design. When the designed system has been completed through implementation and testing, system designers run an error-free version of the software system according to their design intention for each usage scenario, as defined in the requirements phase. In this step, the designer’s actions within the system are detected by sensors mounted on a mobile phone and categorized and logged according to type. The information extracted from the stored action logs is converted into a state diagram. Reflecting the designer’s intentions, the model created through this process is called the “expected activity model,” and is shown in Figure 3. The appropriate time to generate the expected activity model is just before the delivery of a mobile application.

The other activity model is called the “real user activity model,” as shown in Figure 3, and is generated from the action logs detected from actual end users, after the delivery of the mobile application. Although the generation method is the same as that for the expected activity model described above, real user activity models require an additional step: The activity models for multiple users are merged into a representative model of overall user perceptions. However, this step may be omitted if the proposed technique is applied to personalization, i.e., individualized characteristics of mobile application usage. Among the different components of the architecture already introduced in the previous section, the *UAMMonitor* generates activity models from the collected action logs, and merges multiple activity models into a representative model when needed. 

By analyzing differences between the expected activity model, which reflects and implements behavioral assumptions in the current system, and the (real) user activity model, which reflects users’ actual experiences with the system, gaps between the currently provided system and the system desired by real users can be identified. These gaps lead to the identification of latent requirements. The *BadSymptomDetector* compares and analyzes the expected activity model and the user activity model. The rule set for analyzing the gap between an expected activity model and a user activity model can be defined differently, depending on which quality attribute is prioritized. In this paper, the usability of the software as determined by the end user, was selected as the first attribute to be analyzed. Thus, the *BadSymptomDetector* used a predefined rule set to automatically detect four types of bad symptoms (unexpected action sequence, unexpected gesture, repeated gesture, and exceeded elapsed time) related to usability, from differences between the expected activity model and the user activity model. 

## 5. An Automatic User Activity Analysis Process with a Case Study for Usability Issue Detection in a Mobile Application

In this section, details of the activities that constitute the user activity analysis method introduced in the previous section are described step by step, together with tangible examples.

### 5.1. Generate Activity Model Using Automated Finite-State Machine

Not all mobile phone user activities are subject to logging. Considering the resources required to store and manage log data in *Local Environment* storage, only elements that affect the usability of an application should be logged. To designate the GUI controls, for which the action logs should be generated, we first used Android Hierarchy Viewers to convert the structure of a given application’s GUI view into a tree form. The GUI controls, for which the user action logs were generated, were then specified by designating IDs for those controls judged to possibly generate usability issues. 

**Figure 4:**
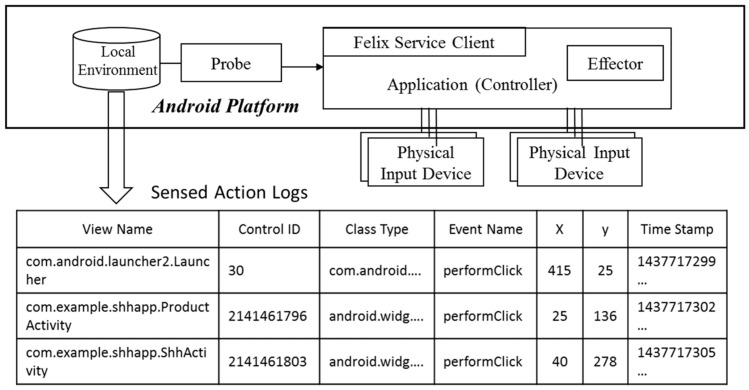
Generation of action logs.

When an event occurs in a monitored GUI element, the *Probe* ported onto the Android platform “sniffs” the event and generates an action log, as schematized in Figure 4, storing it in *Local Environment* storage. An action log contains information such as *Time Stamp*, *Event Name*, *Control ID*, *Class Type, and View Name.* Although the information about which event occurred when at specific (*x*, *y*) coordinates on the screen in an actual Android phone can be obtained through *Probe* event sniffing, the information on the control ID that targets the event, cannot be grasped through the *Probe*. To do so, we used Monkeyrunner, which is an Android macro. Since Monkeyrunner can determine when and which events occurred on an Android phone, we completed an action log, as shown in the table at the bottom of Figure 4, by combining the information with log records obtained through the *Probe* ported onto the Android platform. 

**Figure 5:**
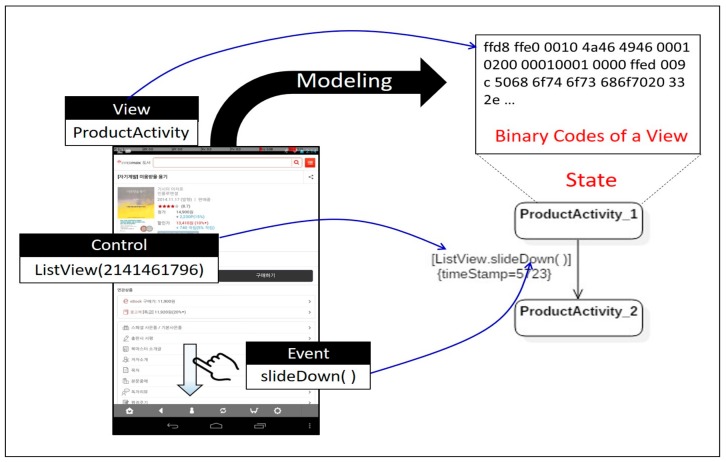
Transition from action log to activity model.

Action logs are saved in *Local Environment* storage, in the form of XML files. The *UAMMonitor*, bound to the *ActivityMonitor* as specified in Figure 1, periodically reads the action logs stored in *Local Environment* storage to generate an activity model. The activity model is expressed as a state diagram, one of UML notations to represent finite state machine (FSM). The state of the screen when a user operates a mobile application is represented by a state, and a transition between states represents an event corresponding to the action taken by a user. The name of each state is composed of the view name on which the event occurred and sequence number. On the transition arrow, the name of the control where the event occurs is denoted as a guard condition with the event name. A sequence diagram allows for designers to extend semantics of a model, by adding new tagged values on the existing symbols. We use the tagged value as a tool to represent the time stamp information, which shows when the event occurred. Information about the ID and control location in the log is not reflected in the activity model, because the information is only used to figure out which control the event occurred on. Figure 5 schematizes a fragment of an activity model generated by the *UAMMonitor* from an action log, indicating that an event called *slideDown( )* occurred in *ListView* control in a view called *ProductActivity*, at the location with coordinates (25, 136), at a time 5252 ms. The *UAMMonitor* reads the action logs stored during the execution of the mobile application in units of one scenario, and then repeats the mapping as shown in Figure 5, to compose activity models in the form of the entire state diagram.

The process for creating a user activity model is the same, irrespective of the kind of user activity model. The only difference is who generates the action logs, and when the action logs are captured. If the action logs are extracted from actual users during the usual running time of a mobile application, the generated activity model will be a user activity model. On the other hand, if an activity model is generated from the action logs of developers before the deployment of a mobile application, it will be an expected activity model.

### 5.2. Merge Multiple User Activity Models into a Representative Real User Activity Model

User activity models are not uniform, because unique characteristics of each individual’s mobile application manipulation are reflected in each model. Accordingly, some parts of individual users’ activity models will be common, and others will vary. The merging process enables the creation of a representative activity model, which includes both common and different user behaviors. In this merging process, it is possible to judge whether slightly different forms of individual models represent the same behavior. Furthermore, minimizing the number of model comparisons to just one comparison, between the merged representative user activity model and the expected activity model, improves the detection of usability-hindering elements. 

**Figure 6:**
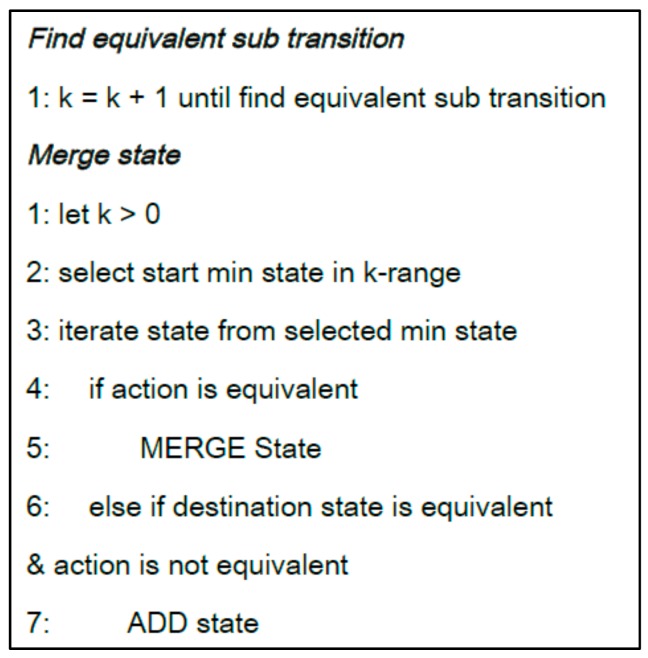
Pseudo-code for Gk-tail algorithm.

The Gk-tail algorithm [25] grasps the equivalent parts among FSMs and merges them into a single model. According to Zhang’s et al. work [26], its weakness is that the predicates automatically generated from the second step are not quite correct. However, this study uses the Gk-tail algorithm to make the user activity model include more user behaviors as possible. Moreover, the bad symptom instances which have lower occurrence rate than a predefined threshold value, will be filtered out in the next step. For this, we analyzed that the issue regarding generation of wrong predicates of the Gk-algorithm is not quite severe in our domain. Hence, we adopted the Gk-algorithm in merging diverse user activity models, into a single representative user activity model. The Gk-tail algorithm compares two different FSM models to equivalent state transitions, and sequentially adds the last state transition of a merged model, indicated by a *k* value, to the FSM to complete the merged model. Figure 6 shows the general steps of the Gk-tail algorithm, described in pseudo-codes. 

**Figure 7:**
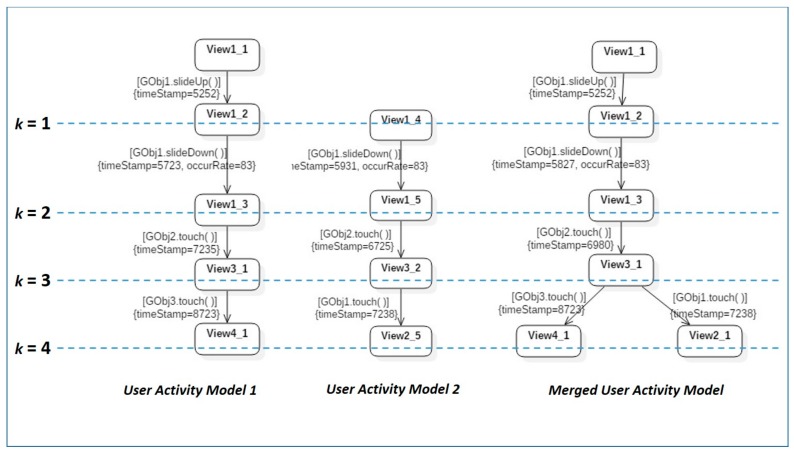
Applying Gk-tail algorithm to merge user activity models.

To more easily understand how we use the Gk-tail algorithm, we will consider the example of merging two different state models (FSMs), as schematized in Figure 7. First, while comparing the state transitions of the two state models, equivalent transitions (which generate the same event on the same GUI object) should be sought. For example, if the *GOjb1.slideDown( )* event and the *GObj2.touch( )* event transitions in Figure 7, are found to be equivalent transitions in the two state models, the *k* value of the first state of the merged state model should be set to 1. It can be seen that the *k* values of state *View1_2* of *user activity model 1* and state *View1_4* of *user activity model 2* in Figure 7, have been set to 1. In cases where the events that caused transitions from the two states are identical, of which the *k* values have been set to 1, to states *View1_3* and *View1_5*, respectively, and the GUI controls where the events occurred are also identical, the *k* value should be increased by 1 each time, until different transitions are found to traverse the state model. In the example shown in Figure 7, we can see that this state model traversed to *k = 3*. This means that the three transitions with *k* values 1 to 3 were equivalent. In this case, states *View1_2*, *View1_3*, and *View3_1* of *user activity model 1*, and states *View1_4*, *View1_5*, and *View3_2* of *user activity model 2*, which can be reached through equivalent transitions, are identified as equivalent states, and therefore merged into states *View1_2*, *View1_3*, and *View3_1* of in the merged user activity model. However, state *View4_1* of *user activity model 1* and state *View2_5* of user *activity model 2,* cannot be merged into the same state, because they are reached through non-equivalent transitions in the two models after *k = 3*. Therefore, two different states that can be reached from state *View3_1* of the merged model are added. Depending on events occurring after state *View3_1* has been reached, state transitions can occur to states *View4_1* and *View2_5*. In the transitions to states *View4_1* and *View2_5*, the probability that each transition actually occurs, i.e., the occurrence rate corresponding to the number of activity models with the transition compared to the total number of activity models, is augmented. The timestamp value of the merged user activity model, is denoted as the average of the values recorded in individual states of multiple user activity models. As such, by applying the Gk-tail algorithm repeatedly, a representative user activity model can be obtained that encompasses multiple activity models extracted from all involved users.

### 5.3. Analyze Differences between Expected Activity Model and Merged Real User Activity Model

When all users’ activity models have been integrated into a representative activity model, this model is compared to an expected activity model constructed from developers’ behavioral assumptions at the time of design. Differences between the two models are analyzed to identify potential bad symptoms, which may hinder the usability of the mobile application. Not all the differences between the two models are necessarily interpreted as bad symptoms. Bad symptoms indicate that the occurrence rates of differences in a representative user activity model are higher than a certain threshold value—that is, cases where a significant majority of users take actions different from expected behaviors. There is no standardized threshold value for judging whether the occurrence rates of differences, between a representative activity model and an expected activity model, are significant. In the literature [2], it was proposed that a GUI that meets 60% of user requirements in a GUI usability test is considered acceptable. Therefore, in this study, a detected difference between a representative user activity model and an expected activity model is subject to bad symptom analysis, if the occurrence rate of the difference is greater than 40%.

To define the types of automatically detectable bad symptoms, we analyzed and grouped characteristics of the GUI issues already raised in open-source mobile application developer communities. As a result, the automatically detectable symptoms were classified into four types:

- **Symptom type 1**: Unexpected action sequence.
- **Symptom type 2**: Unexpected gesture.
- **Symptom type 3**: Repeated gesture.
- **Symptom type 4**: Exceeded elapsed time.

**Figure 8:**
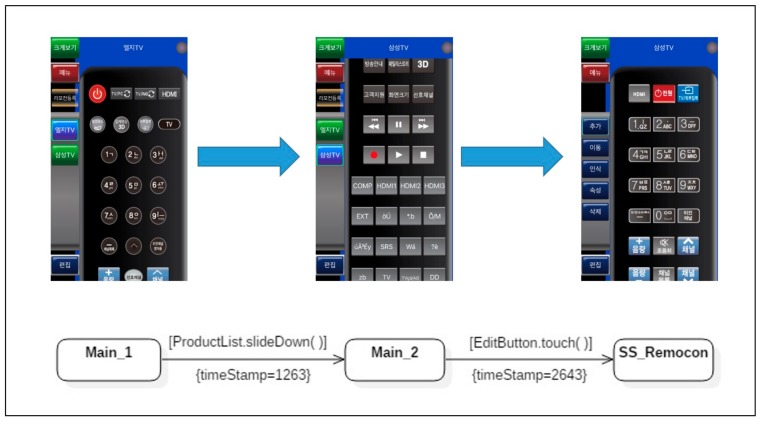
An expected activity model of MyRemocon.

For a more tangible understanding of the symptom types identified, a fragmentary example using MyRemocon, an application provided via Google Play, is presented according to each type. Before presenting the examples, Figure 8 shows an expected activity model, which is a sequence through which the *slideDown( )* action is executed on the object *ProductList,* followed by the *touch( )* action on the object *EditButton*. Although the selected transitions depicted in Figure 8 are very simple, there can exist actual user activity models which contain all four defined symptom types. The details for detecting each bad symptom are described below.

**Figure 9:**
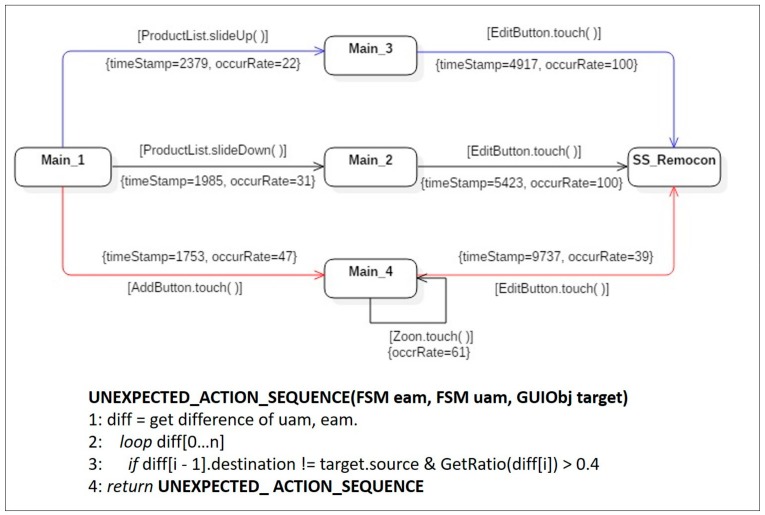
An instance of a user activity model, including bad symptom type 1.

**Symptom Type 1: Unexpected Action Sequence.** The expected activity model in Figure 8, contains only a singular state transition sequence, *Main_1* → *Main_2* → *SS_Remocon*. However, two unexpected action sequences, *Main_1* → *Main_3* → *SS_Remocon* and *Main_1* → *Main_4* → *SS_Remocon*, appear in the merged user activity model in Figure 9. The occurrence rate of the transition *Main_1* → *Main_3* was 22%, which means that 22% (occurRate = 22) of the users followed the sequence. Thus, the sequence *Main_1* → *Main_3* → *SS_Remocon* was neglected as a minor difference because the occurrence rate was less than the threshold (40%), judged to be a meaningful symptom in terms of usability. On the other hand, the action sequence *Main_1* → *Main_4* → *SS_Remocon*, which was not defined in the expected activity model, but was detected in the actual user activity model, was analyzed as an *unexpected action sequence* corresponding to symptom type 1 because the occurrence rate of the first transition *Main_1* → *Main_4* was 47%, 7% greater than the threshold. The unexpected action sequence *Main_1* → *Main_4* → *SS_Remocon* should therefore be registered as a candidate for latent requirements and should be reviewed for potential implementation in the next system revision. 

The algorithms used to detect the bad symptom type 1, are shown in pseudo-codes in Figure 9. An expected activity model (eam) and a merged representative user activity model (uam), were used as input parameters to identify differences (diff) existing between the two models.

**Figure 10:**
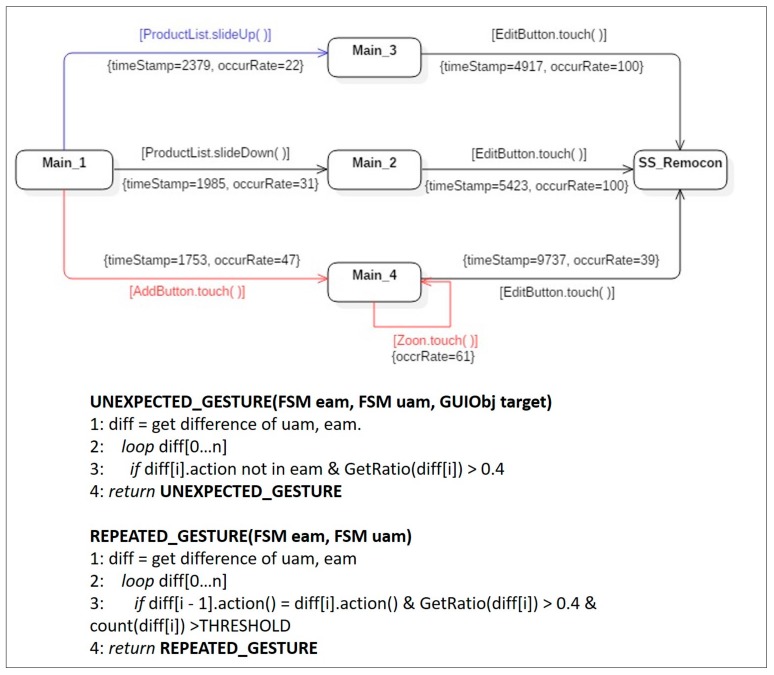
An instance of a user activity model, including bad symptom type 2 and type 3.

**Symptom Type 2: Unexpected Gesture.** As described by the expected activity model in Figure 8, two events were predicted by designers, namely the *slideDown( )* action that occurs in the object *ProductList* and the *touch( )* action that occurs in the object *EditButton*. However, the merged user activity model in Figure 10 detected that users undertook gestures, such as *AddBtton.touch( )*, *ProductList.slideUp( )*, and *Zoom.touch( )*, which were not defined in the expected activity model. Thus, although the application seemed to lead users to use the gesture, the action corresponding to that gesture had not been implemented in the application. However, according to the 40% threshold for bad symptom detection, the *slideUp( )* gesture detected in the *ProductList* control was disregarded because the occurrence frequency was only 22%. Two *touch( )* gestures detected in the *Zoom* control and the *AddButton* control, for which the occurrence frequencies were over 40%, were regarded as symptom type 2, *unexpected gesture*. In the construction of the next version of the MyRemocon application, implementation of the missed touch gesture on the *Zoom* control and the *AddButton* control should be discussed. 

**Symptom Type 3: Repeated Gesture**. The *Zoom.touch( )* gesture was already analyzed as an *unexpected gesture* (symptom type 2) above; here, the *Zoom.touch( )* gesture was identified as an action in the reflexive transition in state *Main_4*. Action in a reflexive transition means that the action can be performed repeatedly, and that the state model can continuously stay in the same state. In terms of usability, this indicates that some users make the same gesture repeatedly to obtain desired services in the same viewing state. Of course, the action is regarded as normal in cases where the same reflexive transition is observed in the expected activity model. However, in this case, the *Zoom.touch( )* gesture was not defined as a reflexive transition action in the expected activity model. Moreover, the occurrence rate was 61%, exceeding the threshold. Thus, the gesture was detected as an instance of bad symptom type 3, *repeated gesture*. This symptom could be resolved by providing a proper help message in the next revision.

**Figure 11:**
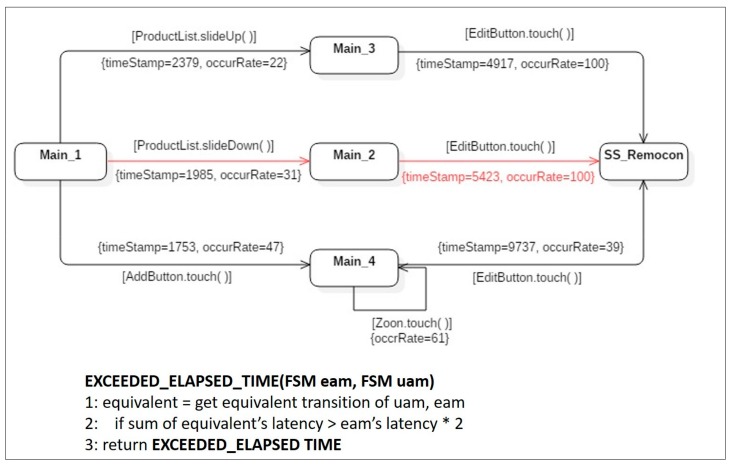
An instance of a user activity model, including bad symptom type 4.

**Symptom Type 4: Exceeded Elapsed Time.** Even equivalent action sequences, included in both an expected activity model and a merged user activity model, can cause a bad symptom if the elapsed time detected in the latter model, exceeds twice the elapsed time for the equivalent transition in the former model. This is the fourth type of bad symptom: *exceeded elapsed time*. In the example shown in Figure 11, the average execution time for actual users to reach the state *SS_Remocon* is 5423 ms. This value was much larger than twice the execution time of 2643 ms of the same transition in the expected activity model. This showed that unlike the designers’ assumptions, actual users took a much longer time to achieve their specific purpose using the mobile application. This result could be interpreted to mean that the current version of the application was not designed to be understood intuitively. To resolve this type of symptom, the design parts requiring more time for users to understand must be identified. The problematic parts of the application’s design should be modified in the next revision, to enhance usability.

## 6. Evaluation

This study utilized two metrics to verify the accuracy and benefits of the proposed method. The first metric was precision: To verify the accuracy of the proposed method, we measured the precision of the automatic detection of usability-hindering elements. Precision and recall are generally used as metrics to measure relevance; however, in the case of detecting latent requirements, it is nearly impossible to define a totally complete set of requirements. Accordingly, we thought it impossible to calculate recall. Thus, we only used precision as a metric to demonstrate the proposed method’s accuracy. To demonstrate the benefits of the proposed method, the second metric selected was the degree of quality improvement to the mobile application when the automatically detected symptoms were resolved.

With these two-selected metrics, we conducted experiments to automatically detect the four types of bad symptoms, from several open-source mobile applications. We selected five applications (see Table 1), for which GUI-related error issues were frequently posted in open-source storage over one year, and for which more than 100 commits were recorded. According to Reference [27], which indicated that the appropriate number of experimental subjects when evaluating GUI usability should be five, we organized three groups of five users who had similar profiles by gender, age group, and app use proficiency. Then, we had them use the selected five applications on mobile devices equipped with the framework introduced in Section 3. The three groups of users executed a total of 174 usage scenarios, selected from the five mobile applications. We applied analysis of variance (ANOVA) [28], a collection of statistical models, to analyze the differences among group means in the sample, to determine whether any kinds of unique characteristics existed in the results of the respective groups. The difference in average values among the groups was 0.318, meaning that there was no statistically significant difference between the groups. Therefore, we did not compare the experimental result values among the groups; instead, we denoted the averaged result of all groups together in Table 1.

The precision values in Table 1, were calculated according to the following calculation Formula (1), redefined to reflect characteristics of the domain for detecting bad symptoms, which hinder the usability of mobile applications.

Precision = TP/(TP + FP)(1)

- **True Positive (TP):** The number of cases where automatically detected symptoms coincide with the usability-related issues posed by actual developers.
- **False Positive (FP)**: The number of cases where automatically detected symptoms were not mentioned in the report of usability-related issues posed by actual developers.

As can be seen from the verification results in Table 1, the average precision of automatic detection of the symptoms of poor usability in two times of experiments was 0.70 and 0.78, respectively. The difference between the results from the two repeated experiments shows the differentiating point of this study, compared with our previous study [5]. In this study, we extended the implementation of the proposed method from a stand-alone typed tool, to a part of a generic self-adaptive software framework. Owing to the extension, as the cycle to detect bad symptoms and resolve them runs again and again in the proposed self-adaptive software architecture, a mobile application can evolve more usable. According to the results in Table 1, the number of TPs detected automatically was 18 in the first round, whereas the number of TPs detected in the second round was significantly reduced to 10. The eight bad symptoms that were detected automatically in the first round, but not in the second round, were automatically resolved through the MAPE cycle implemented by the proposed self-adaptive software framework. In the experimental results of the other four mobile applications, the decrease in the number of TPs due to the implemented self-adaptation cycle in the proposed architecture was equally observed. Even without considering the gradual improvement of precision due to self-adaptation, the results in Table 1 mean that at least 70% of usability-related issues detected by developers through repetitive tests, could be automatically detected when the proposed method was applied. This in turn suggested that the proposed method has the potential to significantly reduce GUI testing time, which amounts to 50–60% [29] of total application development time.

The second metric, measured the degree of quality improvement to mobile applications when automatically detected symptoms (TP in the round 1) were resolved. Table 2 shows the degree to which users’ probability of success was improved through symptom resolution, as they implemented target usage scenarios using the mobile application. Table 2 shows the degree to which the time needed to implement the scenarios was reduced by correcting the mobile application’s bad symptoms, in turn reflecting the usability-inhibiting elements of the mobile application. We measured changes in the probability that users would achieve the final goals of the usage scenarios, as well as how much time it took users to perform each scenario, before and after resolution of the automatically detected bad symptoms. Both measures demonstrated usability improvement after resolving bad symptoms.

According to the experimental results in Table 2, the success rate of users’ implementation of usage scenarios in the mobile applications with potentially bad symptoms was 58% on average, whereas the success rate was on average 80%, when the symptoms were resolved in the next version of the mobile application, showing an improvement of around 21%, compared to the previous version. In addition, the average time needed to execute each scenario using the application before the detection and resolution of bad symptoms was 5125.4 ms, whilst the average time taken to do so using the revised mobile application was 4071.4 ms, demonstrating a reduction of 1082.4 ms. While usability improvements to the mobile application reflect a certain amount of learning from the re-execution of the same scenarios, it is reasonable to suggest that most usability improvements arose from applying the proposed automatic usability analysis method, for detecting and resolving usability issues.

## 7. Conclusions and Future Work

In IoT environments, whilst some change requests are explicitly issued by users, various environmental changes or dynamic configuration changes among participating devices can cause latent requirements, which are difficult for users to express. Thus, to ensure system quality, systems must possess the ability to analyze user activities and detect user contexts, from the results of that analysis. With such a background, this study proposed a method for analyzing user activities conducted on applications operated on Android platform-based mobile phones, and for automatically detecting symptoms that hinder usability. Most of the method’s steps, with the exception of the algorithm used to identify symptoms by issue, are general approaches applicable to the automatic detection of a multitude of bad symptoms related to quality attributes. However, to enable the evaluation of the effectiveness of the method and framework in more tangible instances, the scope of application of the proposed method in this study was limited to usability issues, which are the most closely related issues to the activity patterns of users, among the diverse quality attributes of mobile applications. 

By limiting the target to usability, we could present quantitative results evaluating the accuracy and benefits of the proposed method, with several open-source mobile applications already in use. In the experiments, the proposed method was applied to five open-source applications actually in use in mobile phones, in which an average of 70% of the symptoms were automatically detected as hindering usability, coinciding with the usability-related issues reported by actual developers. Given that the percentage of time spent in GUI testing is 50–60% of the total development time, it can be expected that the overall development time will decrease by applying the proposed method. It was also confirmed that when the automatically detected symptoms were repaired in the next revision, the users benefited from both a higher probability (+21%) of achieving their service goals, and from a reduction in the time (−1082.4 ms) needed to achieve these goals. 

However, some additional follow-up studies must be preceded to apply these benefits in daily life. The proposed method for an automatic analysis of activity model was implemented as part of a self-adaptive framework we developed ourselves. Accordingly, we could detect bad symptoms of mobile applications on Android-based mobile devices on which the proposed framework had already been installed. To extend the proposed method to normal Android-based devices, a whole cycle, including planning and execution phases, in the proposed self-adaptive framework, should be completed. We are currently working on identifying a general set of rules for self-adaptation, and a generic mechanism for executing the automatically generated self-adaptation plans. Another concern in our future work, is the optimization of the size of action logs located in a local mobile device. We are searching for an optimal spot, between the size of the action logs and the amount of network overload, for sending log data by implementing tests in various environmental contexts.

## Figures and Tables

**Table 1:** Precision of symptom detection.

App Name	Round 1			Round 2		
	TP	FP	Precision	TP	FP	Precision
IMSI	9	3	0.75	6	2	0.75
News-android	18	8	0.69	10	3	0.77
MyRemocon	8	4	0.66	5	2	0.71
open-key	5	2	0.71	2	1	0.67
Shhapp	4	2	0.67	2	0	1.00
Average	8.80	3.80	0.70	5.00	1.60	0.78

**Table 2:** Comparison of efficiency: application with vs. without bad symptoms (**a**) Comparison of scenario success rates before and after resolving bad symptoms, (**b**) Comparison of time taken to perform usage scenarios before and after resolving bad symptoms.

	IMSI #59	News-Android #23	MyRemocon #157	Open-Key #54	Shhapp #47	Average
(**a**)						
Success rate before resolving	61%	68%	37%	71%	54%	58%
Success rate after resolving	84%	74%	86%	88%	66%	80%
Changes in success rates	+23%	+6%	+49%	+17%	+12%	+21%
(**b**)						
Time taken before resolution	4975 ms	5941 ms	7378 ms	3259 ms	4074 ms	5125.4 ms
Time taken after resolution	3157 ms	5324 ms	5349 ms	3117 ms	3268 ms	4043 ms
Changes in time taken	−1818 ms	−617 ms	−2029 ms	−142 ms	−806 ms	−1082.4 ms
